# Supplementary material for: Modulation of EZH2 Activity Induces an Antitumoral Effect and Cell Redifferentiation in Anaplastic Thyroid Cancer
Source: Int J Mol Sci. 2023 Apr 26;24(9):7872. doi: 10.3390/ijms24097872 (PMC10178714; doi:10.3390/ijms24097872)
Supplement: Supplementary file 1 [file ijms-24-07872-s001.zip › ijms-2283824-supplementary.pdf]

International Journal of *Molecular Sciences*

MDPI

Supplementary Material

Table S1. Validation of CRISPR/Cas9-mediated *EZH2* gene editing with sgRNA18 in SW1736-ClA using the SeqScreener Gene App

<table>
<tr><th>Frequence</th><th>DNA Sequence</th><th>Indel Size</th></tr>
<tr><td>11.91%</td><td>GAAGCGTGTAAAATCAGAGTACATG---------------|---AGGTTCAGACGAGCTGATGAAGTAAAGG</td><td>-18</td></tr>
<tr><td>7.28%</td><td>GAAGCGTGTAAAATCAGAGTACATGCGACTGAG-------|---------------GCTGATGAAGTAAAGG</td><td>-22</td></tr>
<tr><td>7.18%</td><td>GAAGCGTGTAAAATCAGAGTACATG---------------|--GAGGTTCAGACGAGCTGATGAAGTAAAGG</td><td>-17</td></tr>
<tr><td>6.54%</td><td>GAAGCGTGTAAAATCAGAGTACATGCGACTGAGACAGCTC|+AAGAGGTTCAGACGAGCTGATGAAGTAAAG</td><td>1</td></tr>
<tr><td>5.89%</td><td>GAAGCGTGTAAAATCAGAGTACATGCGACTGAGACAGCT-|AAGAGGTTCAGACGAGCTGATGAAGTAAAGG</td><td>-1</td></tr>
<tr><td>5.28%</td><td>GAAGCGTGTAAAATCAGAGTACATGCGACTGAGACA----|---------------GCTGATGAAGTAAAGG</td><td>-19</td></tr>
<tr><td>4.21%</td><td>GAAGCGTGTAAAATCAGAGTACATGCGACTGAGACAGCT-|---------------GCTGATGAAGTAAAGG</td><td>-16</td></tr>
<tr><td>4.08%</td><td>GAAGCGTGTAAAATCAGAGTACATGCGACTGAGACAGCTC|+++++++++++++++++++AAGAGGTTCAGA</td><td>19</td></tr>
<tr><td>4.01%</td><td>GAAGCGTGTAAAATCAGAGTACATG---------------|---------AGACGAGCTGATGAAGTAAAGG</td><td>-24</td></tr>
<tr><td>3.92%</td><td>GAAGCGTGTAAAATCAGAGTACATGCGACTGAGACAGCTC|+++++++++++++AAGAGGTTCAGACGAGCT</td><td>13</td></tr>
<tr><td>3.31%</td><td>GAAGCGTGTAAAATCAGAGTACATG---------------|------------CGAGCTGATGAAGTAAAGG</td><td>-27</td></tr>
<tr><td>2.94%</td><td>GAAGCGTGTAAAATCAGAGTACATGCGACTGAGACAGCTC|---AGGTTCAGACGAGCTGATGAAGTAAAGG</td><td>-3</td></tr>
<tr><td>2.93%</td><td>GAAGCGTGTAAAATCAGAGTACATGCGACTGAGACAGCT-|-AGAGGTTCAGACGAGCTGATGAAGTAAAGG</td><td>-2</td></tr>
<tr><td>2.58%</td><td>GAAGCGTGTAAAATCAGAGTACATGCGACTGAGACAGCTC|AAGAGGTTCAGACGAGCTGATGAAGTAAAGG</td><td>0</td></tr>
<tr><td>2.40%</td><td>GAAGCGTGTAAAATCAGAGTACATGCGACTGAGAC-----|------TTCAGACGAGCTGATGAAGTAAAGG</td><td>-11</td></tr>
<tr><td>2.13%</td><td>GAAGCGTGTAAAATCAGAGTACATGCGACTGAGACAGCTC|++++++++++++++++AAGAGGTTCAGACGA</td><td>16</td></tr>
<tr><td>2.00%</td><td>GAAGCGTGTAAAATCAGAGTACATGCGACTGAG-------|--------------AGCTGATGAAGTAAAGG</td><td>-21</td></tr>
<tr><td>1.79%</td><td>GAAGCGTGTAAAATCAGAGTACATGCGACTGAGACAGCTC|+++++++++AAGAGGTTCAGACGAGCTGATG</td><td>9</td></tr>
<tr><td>1.66%</td><td>GAAGCGTGTAAAATCAGAGTACATG---------------|--------CAGACGAGCTGATGAAGTAAAGG</td><td>-23</td></tr>
<tr><td>1.56%</td><td>GAAGCGTGTAAAATCAGAGTACATGCGACTGAGACAGCTC|++++++AAGAGGTTCAGACGAGCTGATGAAG</td><td>6</td></tr>
<tr><td>1.56%</td><td>GAAGCGTGTAAAATCAGAGTACATGCGACTGAGAC-----|------------CGAGCTGATGAAGTAAAGG</td><td>-17</td></tr>
<tr><td>1.49%</td><td>GAAGCGTGTAAAATCAGAGTACATGCGACTGAGACAGCTC|+++++++++++++++++AAGAGGTTCAGACG</td><td>17</td></tr>
<tr><td>1.22%</td><td>GAAGCGTGTAAAATCAGAGTACATGCGACTGAGACAGCTC|-------------GAGCTGATGAAGTAAAGG</td><td>-13</td></tr>
<tr><td>1.16%</td><td>GAAGCGTGTAAAATCAGAGTACATG---------------|------TTCAGACGAGCTGATGAAGTAAAGG</td><td>-21</td></tr>
<tr><td>1.14%</td><td>GAAGCGTGTAAAATCAGAGTACATGCGACTGAGACAGCTC|+++AAGAGGTTCAGACGAGCTGATGAAGTAA</td><td>3</td></tr>
<tr><td>1.10%</td><td>GAAGCGTGTAAAATCAGAGTACATG---------------|-----------ACGAGCTGATGAAGTAAAGG</td><td>-26</td></tr>
<tr><td>1.02%</td><td>GAAGCGTGTAAAATCAGAGTACATGCGACTGAGACAGCTC|+++++++++++++++AAGAGGTTCAGACGAG</td><td>15</td></tr>
<tr><td>1.00%</td><td>GAAGCGTGTAAAATCAGAGTACATG---------------|----------GACGAGCTGATGAAGTAAAGG</td><td>-25</td></tr>
<tr><td>0.89%</td><td>GAAGCGTGTAAAATCAGAGTACATGCGACTGAGACAGCT-|--GAGGTTCAGACGAGCTGATGAAGTAAAGG</td><td>-3</td></tr>
<tr><td>0.87%</td><td>GAAGCGTGTAAAATCAGAGTACATG---------------|--------------AGCTGATGAAGTAAAGG</td><td>-29</td></tr>
<tr><td>0.84%</td><td>GAAGCGTGTAAAATCAGAGTACATGCGACTGAGACAGCTC|+++++++AAGAGGTTCAGACGAGCTGATGAA</td><td>7</td></tr>
<tr><td>0.83%</td><td>GAAGCGTGTAAAATCAGAGTACATGCGACTGAGACAGCTC|++AAGAGGTTCAGACGAGCTGATGAAGTAAA</td><td>2</td></tr>
<tr><td>0.79%</td><td>GAAGCGTGTAAAATCAGAGTACATGCGACTGAGACAGCTC|++++++++++AAGAGGTTCAGACGAGCTGAT</td><td>10</td></tr>
<tr><td>0.77%</td><td>GAAGCGTGTAAAATCAGAGTACATG---------------|AAGAGGTTCAGACGAGCTGATGAAGTAAAGG</td><td>-15</td></tr>
<tr><td>0.69%</td><td>GAAGCGTGTAAAATCAGAGTACATGCGACTGAGACAGCTC|++++++++++++++++++++AAGAGGTTCAG</td><td>20</td></tr>
<tr><td>0.52%</td><td>GAAGCGTGTAAAATCAGAGTACATGCGACTGAGAC-----|-------TCAGACGAGCTGATGAAGTAAAGG</td><td>-12</td></tr>
<tr><td>0.51%</td><td>GAAGCGTGTAAAATCAGAGTACATGCGACTGAGACAGCTC|++++++++++++AAGAGGTTCAGACGAGCTG</td><td>12</td></tr>
</table>

Table S2. Validation of CRISPR/Cas9-mediated *EZH2* gene editing with sgRNA18 in SW1736-ClC using the SeqScreener Gene App

Frequence	DNA Sequence	Indel Size
30.39%	GAAGCGTGTAAAATCAGAGTACATGCGACTGAG-------|--GAGGTTCAGACGAGCTGATGAAGTAAAGG	-9
20.26%	GAAGCGTGTAAAATCAGAGTACATGCGACTGAG-------|----GGTTCAGACGAGCTGATGAAGTAAAGG	-11
13.28%	GAAGCGTGTAAAATCAGAGTACATGCGACTGAG-------|------TTCAGACGAGCTGATGAAGTAAAGG	-13
13.25%	GAAGCGTGTAAAATCAGAGTACATGCGACTGAGACAGCT-|AAGAGGTTCAGACGAGCTGATGAAGTAAAGG	-1
10.24%	GAAGCGTGTAAAATCAGAGTACATGCGACTGAG-------|-----GTTCAGACGAGCTGATGAAGTAAAGG	-12
7.29%	GAAGCGTGTAAAATCAGAGTACATGCGACTGAGA------|----GGTTCAGACGAGCTGATGAAGTAAAGG	-10
2.26%	GAAGCGTGTAAAATCAGAGTACATGCGACTGAGACA----|AAGAGGTTCAGACGAGCTGATGAAGTAAAGG	-4
1.67%	GAAGCGTGTAAAATCAGAGTACATGCGACTGAG-------|AAGAGGTTCAGACGAGCTGATGAAGTAAAGG	-7
1.36%	GAAGCGTGTAAAATCAGAGTACATGCGACTGAGACAGCTC|AAGAGGTTCAGACGAGCTGATGAAGTAAAGG	0

Table S3. Validation of CRISPR/Cas9-mediated *EZH2* gene editing with sgRNA18 in SW1736-ClE using the SeqScreener Gene App

Frequence	DNA Sequence	Indel Size
17.22%	GAAGCGTGTAAAATCAGAGTACATGCGACTGAG-------|-------TCAGACGAGCTGATGAAGTAAAGG	-14
14.76%	GAAGCGTGTAAAATCAGAGTACATGCGACTGAGA------|----GGTTCAGACGAGCTGATGAAGTAAAGG	-10
14.12%	GAAGCGTGTAAAATCAGAGTACATGCGACTGAGACA----|AAGAGGTTCAGACGAGCTGATGAAGTAAAGG	-4
14.04%	GAAGCGTGTAAAATCAGAGTACATGCGACTGAG-------|AAGAGGTTCAGACGAGCTGATGAAGTAAAGG	-7
10.46%	GAAGCGTGTAAAATCAGAGTACATGCGACTGAGACA----|-AGAGGTTCAGACGAGCTGATGAAGTAAAGG	-5
8.83%	GAAGCGTGTAAAATCAGAGTACATGCGACTGAGACAGCTC|AAGAGGTTCAGACGAGCTGATGAAGTAAAGG	0
8.67%	GAAGCGTGTAAAATCAGAGTACATGCGACTGAG-------|------TTCAGACGAGCTGATGAAGTAAAGG	-13
3.97%	GAAGCGTGTAAAATCAGAGTACATGCGACTGAGAC-----|--GAGGTTCAGACGAGCTGATGAAGTAAAGG	-7
3.91%	GAAGCGTGTAAAATCAGAGTACATGCGACTGAG-------|----GGTTCAGACGAGCTGATGAAGTAAAGG	-11
2.48%	GAAGCGTGTAAAATCAGAGTACATGCGACTGAG-------|-AGAGGTTCAGACGAGCTGATGAAGTAAAGG	-8
1.55%	GAAGCGTGTAAAATCAGAGTACATGCGACTGAG-------|--GAGGTTCAGACGAGCTGATGAAGTAAAGG	-9

Table S4. List of potential off-target genes of SgRNA18

Genomic localization	Nº of mismatches	Gene symbol	Observation	Sequence (including mismatches)
chr20:1180923	3	*TMEM74B*	Exonic	CCCaTTGAGCTGTCTCAtgCGCA
chr4:188610246	3	*LINC01060*	Intergenic	CCTCTTGAGaTGaCTCAGTCtCA
chr7:1979269	3	*MAD1L1*	Intronic	CCCCTgGAGCTGTCTCAGTCcCc
chr8:120083190	3	*COL14A1*	Intergenic	CCTCTTtgGCTGTCTCAGTCtCA

Table S5. Thyroid cancer cell lines and culture conditions

Histology	Cell line	Genetic driver	Culture medium*
PTC	BCPAP	$BRAF^{V600E}$	DMEM + 10% FBS
ATC	KTC2	$BRAF^{V600E}$	RPMI1640 + 5% FBS
	SW1736	$BRAF^{V600E}$	RPMI1640 + 10% FBS
	8305C	$BRAF^{V600E}$	RPMI1640 + 10% FBS

* FBS – Fetal bovine serum.

Table S6. List of primers used in this study

	Sequence 5'-3' *	Experiment
sgRNA 7 Fw	**CACCG**ACACGCTTCCGCCAACAAAC	cloning in PX459 plasmid
sgRNA 7 Rv	**AAAC**GTTTGTTGGCGGAAGCGTGT**C**	cloning in PX459 plasmid
sgRNA 18 Fw	**CACCG**TGCGACTGAGACAGCTCAAG	cloning in PX459 plasmid
sgRNA 18 Rv	**AAAC**CTTGAGCTGTCTCAGTCGCA**C**	cloning in PX459 plasmid
sgRNA 25 Fw	**CACCG**CAGACGAGCTGATGAAGTAA	cloning in PX459 plasmid
sgRNA 25 Rv	**AAAC**TTACTTCATCAGCTCGTCTG**C**	cloning in PX459 plasmid
EZH2 FW	ATTTAACCATGCAGCACAAATG	DNA sequencing of target region
EZH2 RV	CAGATCAAGAACCTAAGCTTCCA	DNA sequencing of target region
TMEM74B Fw	TCGAGTCAATCCGGACACAGT	sg18 off-targeting DNA sequencing
TMEM74B Rv	GAGCTCAGTTTAAAACCCCAGG	sg18 off-targeting DNA sequencing
RPL19 FW	TCTCATGGAACACATCCACAA	qPCR
RPL19 RV	TGGTCAGCCAGGAGCTTCTT	qPCR
CDH1 FW	TACCCTGGTGGTTCAAGCTG	qPCR
CDH1 RV	ACCTGACCCTTGTACGTGGT	qPCR
ZEB1 FW	GATGACCTGCCAACAGACCA	qPCR
ZEB1 RV	GCCCTTCCTTTCCTGTGTCA	qPCR
ZEB2 FW	AGTGTGCCCAACCATGAGTC	qPCR
ZEB2 RV	TCCTTCATTTCTTCTGGACCATC	qPCR
NIS FW	AGTACATTGTAAGCCACGATGCTGTA	qPCR
NIS RV	CGGTCACTTGGTTCAGGATGA	qPCR
TG FW	CCTGCTGGCTCCACCTTGTTT	qPCR
TG RV	CCTTGTTCTGAGCCTCCCATCGTT	qPCR
TPO FW	ACGCCTCTGCGAGGTGC	qPCR
TPO RV	TGCAAATCACCGTCGAGGT	qPCR
TSHR FW	CCTTCACCTCACACGGGCT	qPCR
TSHR RV	TGCTCTCATTACACATCAAGGACTC	qPCR
NKX2-1 FW	CAGCCTGTCCCACCTGAACT	qPCR
NKX2-1 RV	ATAGCAAGGTGGAGCAGGACAT	qPCR
PAX8 FW	GGCATGGTGGCAGGAAGT	qPCR
PAX8 RV	GCGCCAGGCCTCGCTGTAGGA	qPCR
FOXE1 FW	TGAGCCAGCGTAGGGACGAAAA	qPCR
FOXE1 RV	CCACCTCCTCCCGTTTACAGAGTA	qPCR
GLIS3 FW	AACGCCCGCTATAAACTGCT	qPCR
GLIS3 RV	CTCGCAACCTTCAAACGTACA	qPCR
EED FW	TGGATTTCTGGCAAAAGATGCT	qPCR
EED RV	TATCGAAGTCGATCCCAGCG	qPCR
SUZ12 FW	GAAGCCGAAAATGGAGCACG	qPCR
SUZ12 RV	GTTCTGGAGTTTCGATGAGACAT	qPCR
EZH2 FW	TACTTGTGGAGCCGCTGAC	qPCR
EZH2 RV	CTGCCACGTCAGATGGTG	qPCR

* FW – forward, RV – reverse

Table S7. List of antibodies and dilutions for protein expression analysis

Antibody	Source	Dilution	Application*
mouse anti-E-Cadherin	Cell signaling	1:1000	WB
mouse anti-N-Cadherin	Sigma	1:500	WB
rabbit anti-b-Catenin (sc-7199)	Santa Cruz	1:1000	WB
rabbit anti-c-Myc (sc-764)	Santa Cruz	1:1000	WB
rabbit anti-Cyclin D1	Cell signaling	1:1000	WB
rabbit anti-EED	Cell signaling	1:1000	WB
rabbit anti-EZH2	Cell signaling	1:1000	WB
rabbit anti-H3	Cell signaling	1:2000	WB
rabbit anti-H3K27me3	Cell signaling	1:1000	WB
rabbit anti-Ki67	Sigma	1:100	IHC
rabbit anti-αSMA	Sigma	1:100	IHC
rabbit anti-ZEB1	Invitrogen	1:1000	WB

* WB – western blot, IHC – immunohistochemistry

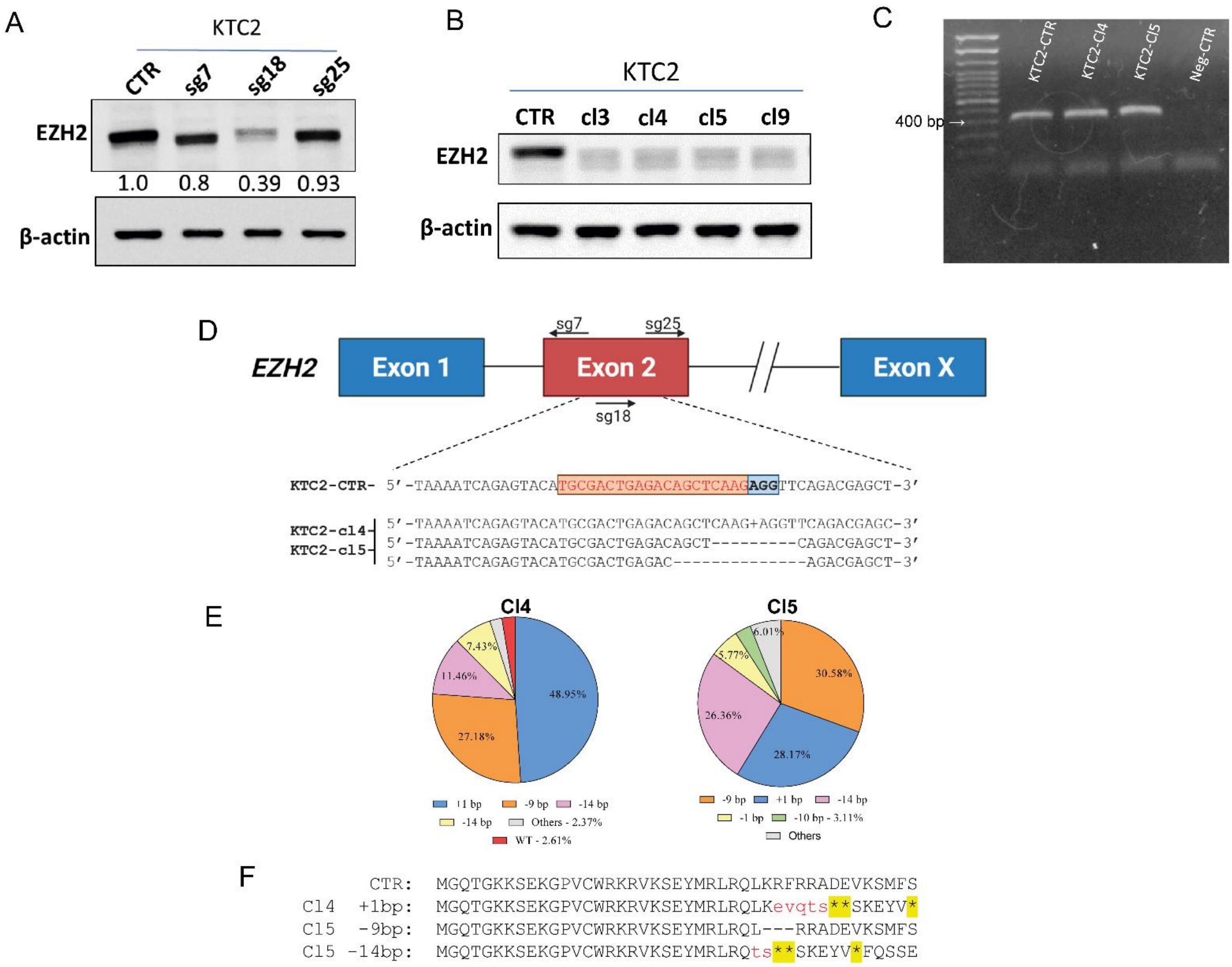

Figure S1. Targeting the *EZH2* gene with CRISPR/Cas9 in the KTC2 cell line. **(A)** EZH2 protein levels in the mixed populations of KTC2 transfected with different sgRNAs. **(B)**. EZH2 protein levels in the clones derived from KTC2 sg18 (cl3, cl4, cl5 and cl9) **(C)**. PCR amplification of sgRNA target region. We loaded in the 1.5% agarose gel the 100bp ladder (Invitrogen) followed by 5uL of a 25uL PCR (using 150 ng of genomic DNA of each clone). **(D).** Genomic DNA sequencing of KTC2 clones Cl4 and Cl5 after CRISPR/Cas9-mediated EZH2 gene editing. sgRNA-guided target regions are highlighted in red and PAM sequence in bold. The main editing events after dsDNA repair resulted in +1 nt insertion in clone 4 (cl4) and 9 nt or 14 nt deletion in clone 5 (cl5). **(E).** Pie chart showing the main gene editing events in cl4 and cl4 after analysis in SeqScreener App. **(F)**. Alignment of amino acids changes after gene editing (nonsense and missense) in cl4 and cl5. Sg: single guide; CTR: control; cl: clone; PAM: protospacer adjacent motif; *: premature stop codon.

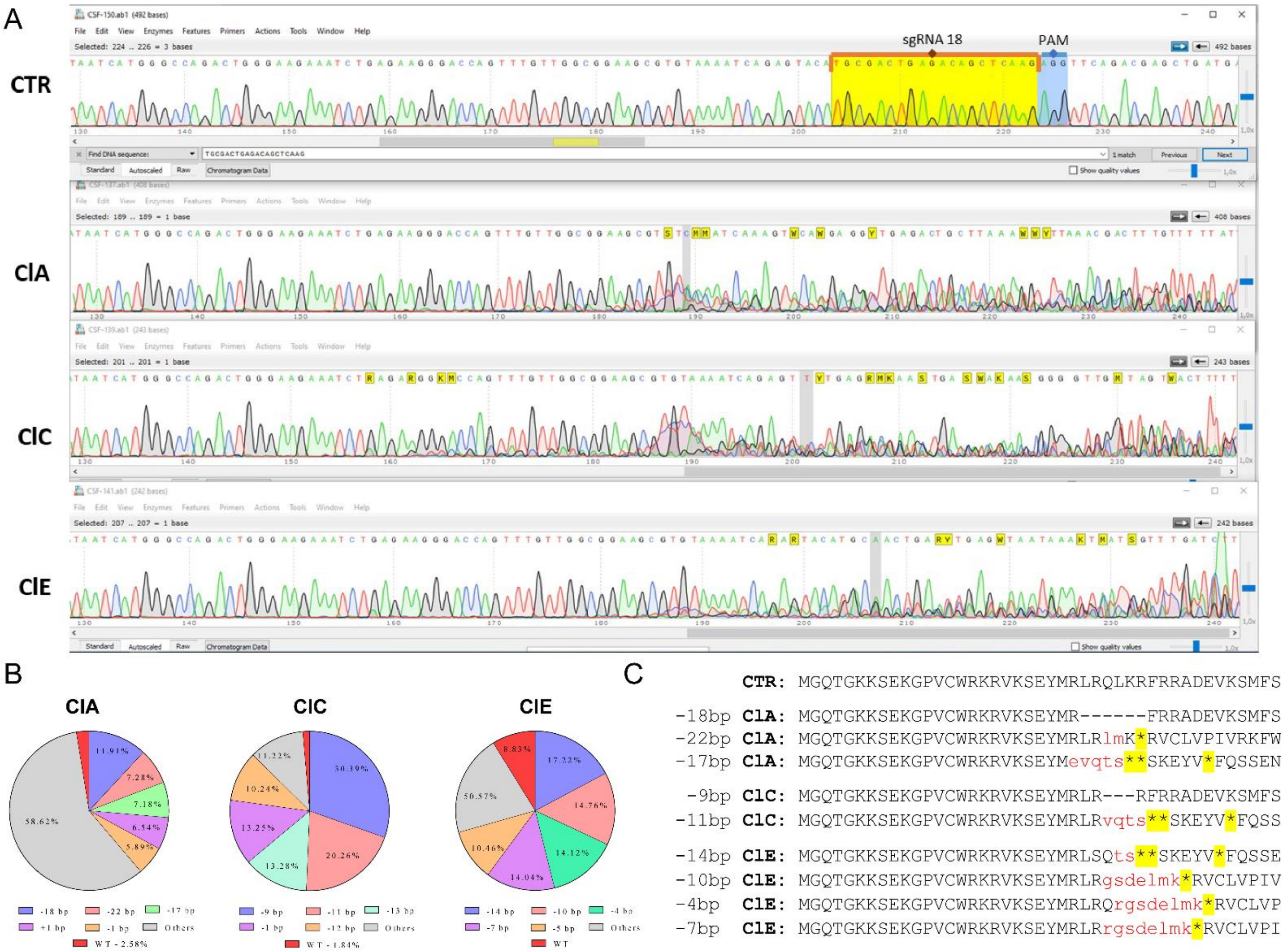

Figure S2. Raw sanger sequencing data alignment for *EZH2* gene in control unedited cells (SW1736 CTR) compared to clones A, C and E. The yellow highlighted region in SW1736-CTR indicates the region targeted by sgRNA18 and PAM sequences are highlighted in blue. R: A or G; Y: C or T; S: C or G; W: A or T; K: G or T; M: A or C. **(B).** Pie chart showing the main gene editing events and their respective frequencies in ClA, ClC and ClE cells after analysis in SeqScreener App. **(C)**. Alignment of amino acids changes after gene editing (nonsense and missense) in ClA, ClC and ClE. Sg: single guide; CTR: control; cl: clone; PAM: protospacer adjacent motif; *: premature stop codon.

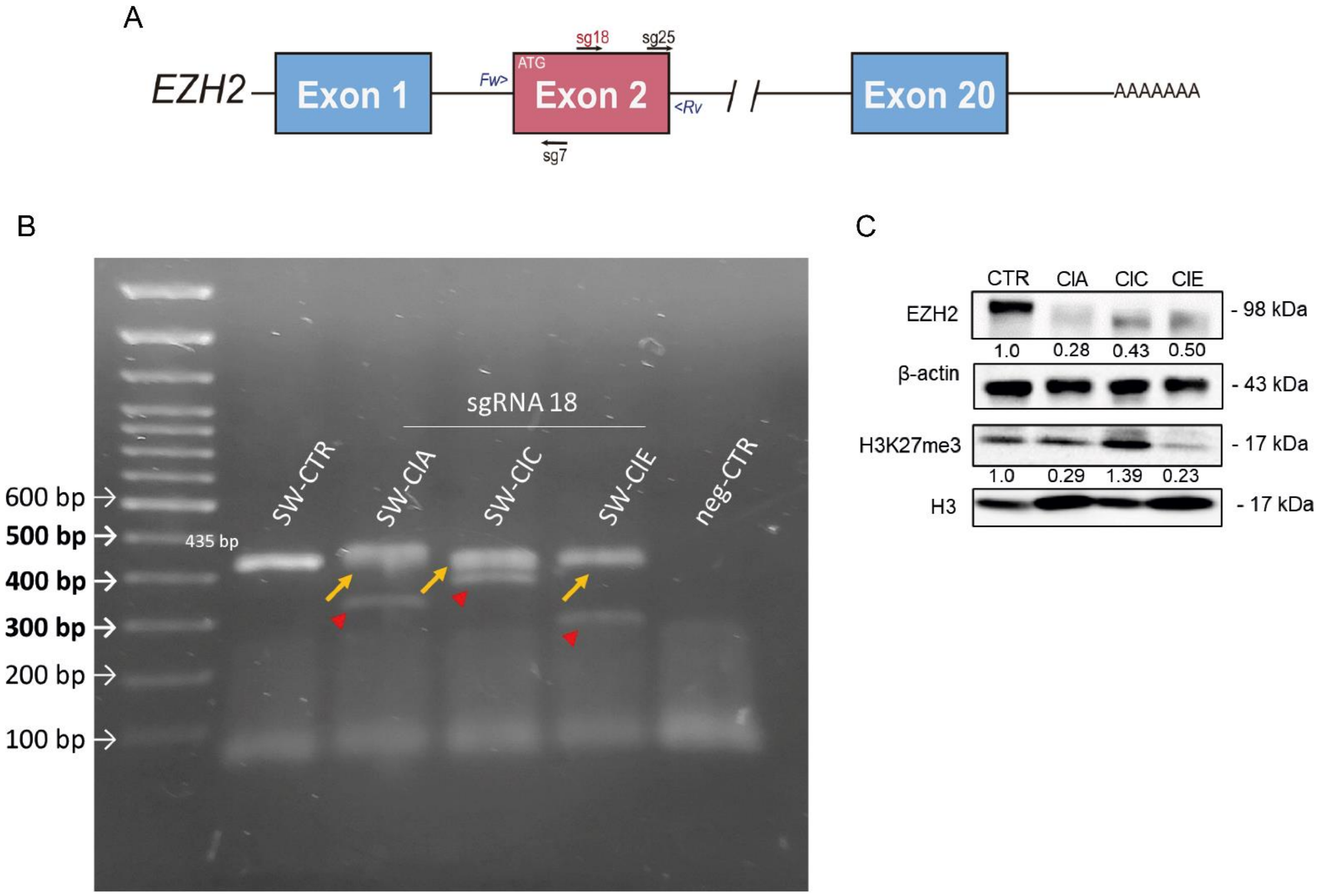

Figure S3. PCR amplification of CRISPR/Cas9-sg18 target region in SW-clones A, C and E using primers flanking *EZH2* exon 2 **(A)**. In the control unedited SW1736, the presence of a single band of 435 bp is expected, while, in the edited clones, we observe different smaller fragments indicating deletions in the agarose gel **(B)**. We loaded in the 1.5% agarose gel the 100bp ladder (Invitrogen) followed by 5uL of a 25uL PCR (using 150 ng of genomic DNA of each clone). In **C**, we show again the protein levels of EZH2 in the clones.

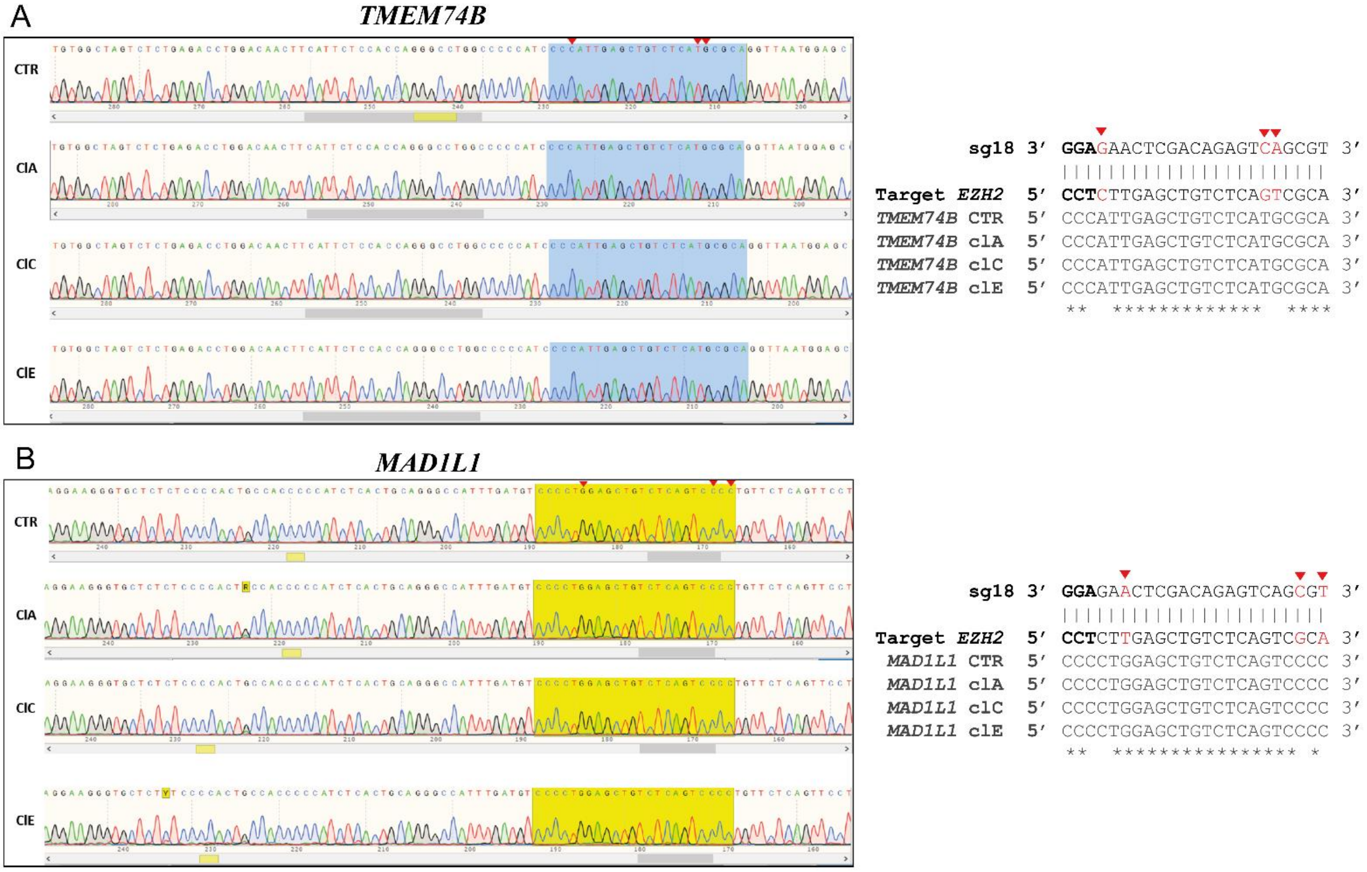

Figure S4. Raw sanger sequencing data alignment for *TMEM74B* and *MAD1L1* genes, predicted off-target coding-genes in the ChopChop algorithm. **(A)** The left panel shows the alignment of samples electropherogram and red arrowhead indicates potential off-target site of sgRNA18 in *TMEM74B* gene. Highlighted in blue, we indicate the potential target of sg18. In the left panel, we aligned sequences of SW1736-CTR and SW1736-edited cells showing no off-targeting in *TMEM74B* gene. **(B)** The left panel shows the alignment of samples electropherogram and red arrowhead indicates potential off-target site of sgRNA18 in *MAD1L1* gene. Highlighted in yellow, we indicate the potential target of sg18. In the left panel, we aligned sequences of SW1736-CTR and SW1736-edited cells showing no off-targeting in *MAD1L1* gene.

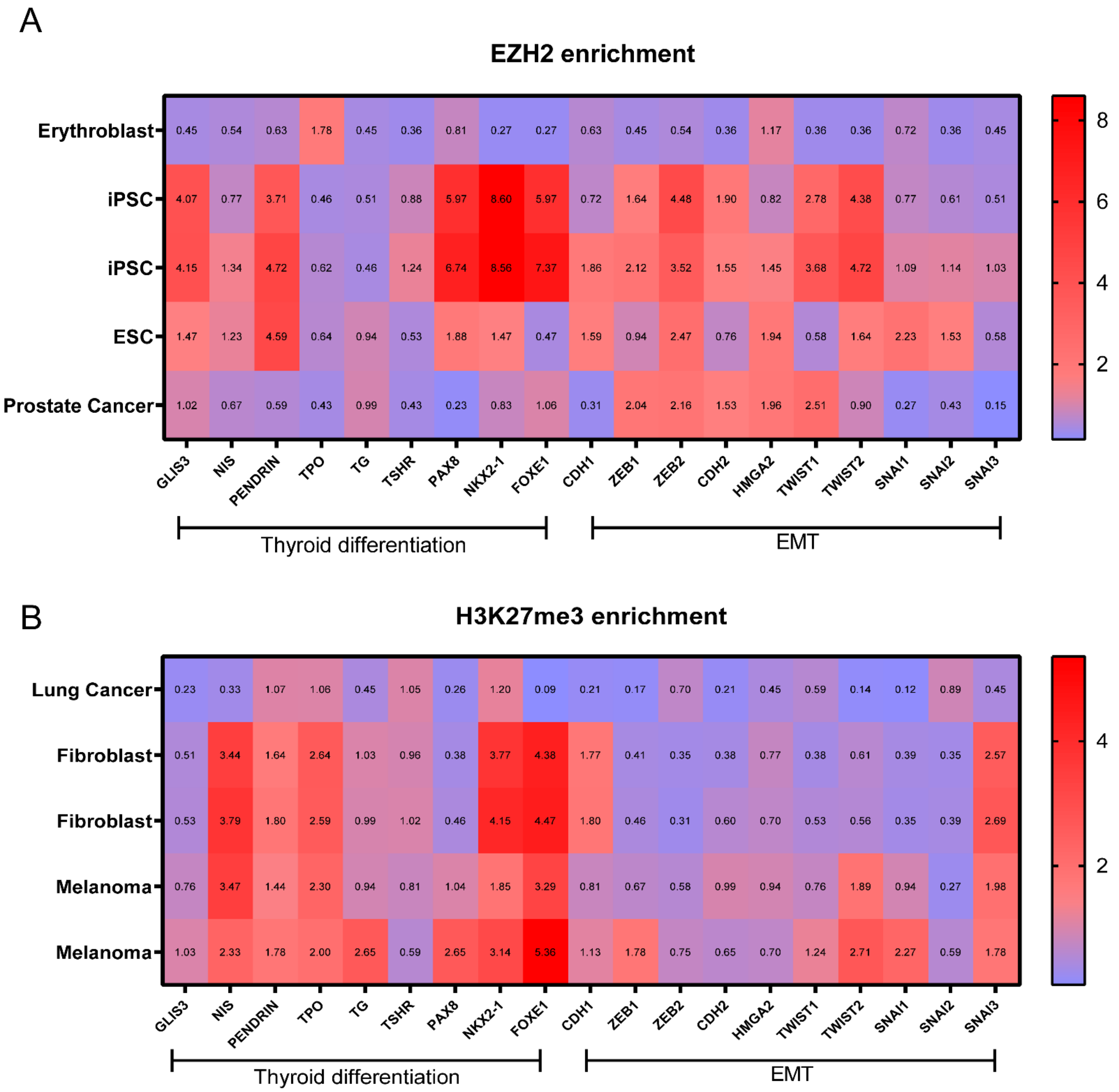

Figure S5. Cistrome data browser analysis of EZH2 **(A)** and H3K27me3 **(B)** binding at thyroid differentiation, EMT and Wnt signaling genes. For EZH2 binding data, the following Cistrome ID studies were utilized: 74684 (Erythroblast), 41794 (iPSC), 41788 (iPSC), 9009 (ESC) and 8664 (Prostate Cancer). For H3K27me3 binding data, the following Cistrome ID studies were utilyzed: 45141 (Lung Cancer), 71329 (Fibroblast), 71328 (Fibroblast), 52394 (Melanoma), 52389 (Melanoma).

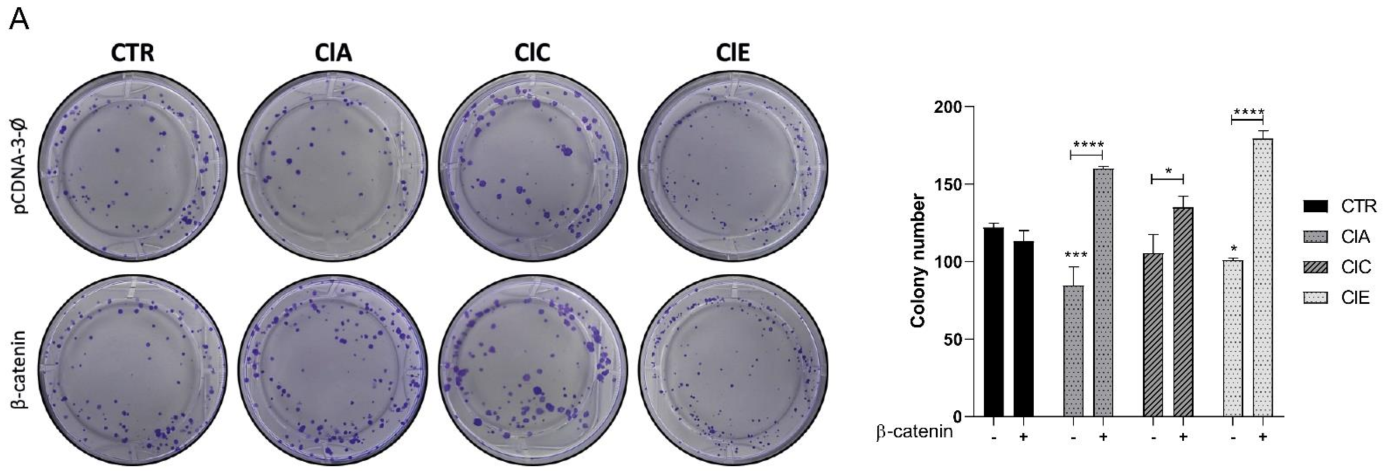

Figure S6. β-catenin rescue assay. β-catenin was transiently overexpressed in SW1736-CTR and SW1736-ClA, SW1736-ClC and SW1736-ClE cells. **(A)** Colony formation assay and quantification of number of colonies in cells transfected with an empty pCDNA (-) or with β-catenin (+). *, $p < 0.05$; ***, $p < 0.001$; ****, $p < 0.0001$ *vs* SW1736-CTR or pCDNA (-) cells.
